# Sensor-Based Channel-Wise Remaining Useful Life Estimation and Multi-Threshold Maintenance Policy for Steel-Cord Conveyor Belts

**DOI:** 10.3390/s26154944

**Published:** 2026-08-05

**Authors:** Ryszard Błażej, Leszek Jurdziak, Aleksandra Rzeszowska

**Affiliations:** Faculty of Geoengineering, Mining and Geology, Wrocław University of Science and Technology, na Grobli 15 St., 50-421 Wroclaw, Poland; ryszard.blazej@pwr.edu.pl (R.B.); leszek.jurdziak@pwr.edu.pl (L.J.)

**Keywords:** steel-cord conveyor belts, sensor-based diagnostics, non-destructive testing, channel-wise health indicators, remaining useful life, condition-based maintenance, predictive maintenance, refurbishment policy, spatial degradation

## Abstract

Steel-cord conveyor belts are usually assessed using average damage indicators calculated for entire belt sections. Although such indicators are useful for general condition assessment, they do not adequately represent spatially non-uniform degradation across the belt width. This paper proposes a sensor-based, channel-wise framework for condition assessment, remaining useful life (RUL) estimation, and maintenance decision support for steel-cord conveyor belts. The approach uses data from consecutive non-destructive diagnostic scans, in which the belt cross-section is represented as a set of active measurement channels. For each channel, local damage density is treated as a health indicator evolving over time. A simple accelerating degradation model is then used to estimate local degradation intensity and channel-wise RUL. Three decision thresholds are introduced to represent entry into the refurbishment window, the end of economically justified refurbishment, and the critical removal condition. In addition to channel-wise RUL, the framework includes cross-sectional indicators such as the fraction of channels exceeding each threshold, the width of compact critical zones, and life-uniformity measures describing the spatial distribution of degradation. A cost-oriented interpretation is also introduced by linking non-uniform cross-sectional degradation with reduced life utilization and increased cost per unit operating time. The applicability of the framework is illustrated using consecutive diagnostic scans from an industrial conveyor belt loop. The results show that localized degradation may govern belt replacement earlier than average indicators suggest. The proposed approach provides a practical basis for condition-based maintenance, refurbishment planning, and future integration with loading-condition analysis and digital-twin-oriented diagnostics.

## 1. Introduction

Steel-cord conveyor belts are among the most critical and expensive components of continuous transport systems used in mining, bulk material handling, and large-scale industrial logistics. Their technical condition directly affects transport continuity, operational safety, production stability, and the total cost of conveyor operation. For this reason, conveyor belt diagnostics, condition monitoring, and maintenance decision support have become important areas of both industrial practice and scientific research. In the broader context of asset management, maintenance strategies are increasingly moving from periodic inspection and reactive replacement towards condition-based maintenance and prognostics [1]. In conveyor belt diagnostics, this transition is also supported by sensor-based condition monitoring and diagnostic data analysis [2]. Within this transition, remaining useful life (RUL) estimation plays an important role because it transforms diagnostic information into a time-oriented basis for planning refurbishment, replacement, and risk-controlled operation [1,3,4,5]. This transition is also consistent with broader condition-based maintenance research, where condition monitoring, inspection planning, replacement decisions and prognosis are treated as connected elements of maintenance management [6]. Recent reviews on predictive maintenance in Industry 4.0 emphasize that condition monitoring, data-driven degradation modelling, remaining useful life estimation and decision support are increasingly integrated into one maintenance process rather than treated as separate tasks [7,8,9].

Recent developments in predictive maintenance and prognostics increasingly rely on sensor data, RUL estimation and digital-twin-oriented decision support, which makes diagnostic information a direct input to maintenance planning rather than only a basis for fault detection [1,10].

Steel-cord belts are usually inspected using non-destructive testing systems capable of detecting and locating damage to the steel-cord core. Recent reviews show that conveyor belt diagnostics may involve magnetic, vision-based, thermographic, ultrasonic, acoustic, vibration-based, radar, and hyperspectral methods, depending on the type of damage and the required diagnostic resolution. For steel-cord belts, magnetic diagnostic systems are particularly important because they allow hidden damage to the load-bearing core to be detected without cutting or dismantling the belt [11]. Consecutive diagnostic scans make it possible not only to identify the current number and location of defects, but also to observe damage development over time [3,11]. Such scan-based diagnostic information can also support condition monitoring and predictive refurbishment of conveyor belts [2]. The growth of longitudinal and transverse damage extents has been analysed separately as a time-dependent process [12]. Recent conveyor-belt-oriented reviews distinguish between surface-oriented monitoring methods, such as computer vision, and non-destructive techniques aimed at internal or hidden damage, including magnetic flux leakage, X-ray, ultrasonic, radio-frequency and terahertz inspection [13,14]. Recent reviews also emphasize that predictive maintenance research increasingly combines data acquisition, data processing, diagnosis, prognosis and decision support within one digital maintenance workflow [15].

A key limitation of many practical assessment procedures is that they rely mainly on average damage indicators calculated for an entire belt section. Such indicators are useful for general condition classification, but they do not fully represent the spatial structure of degradation. In real conveyor operation, belt degradation is rarely uniform. A belt loop consists of sections and splices with different ages, repair histories, and operating conditions, and even within a single belt section the steel-cord core may degrade differently along the belt length and across the belt width [11,12,16]. This non-uniformity is particularly important in loading zones, where chute geometry, impact of large lumps, local cover wear, asymmetric material flow, and non-uniform acceleration of bulk material may concentrate damage in selected transverse zones of the belt [11,12,16].

As a result, some parts of the belt cross-section may lose their useful life much faster than others. In such cases, the actual end of useful life of a belt section may be governed not by the average condition of the entire belt width, but by the most degraded local zone. This creates a fundamental diagnostic and prognostic problem: two belt sections may have the same average damage density, while one of them is uniformly degraded and the other contains a compact critical zone. From the point of view of operational risk, refurbishment feasibility, and replacement planning, these two cases are not equivalent [2,3,16]. Therefore, average indicators may mask local degradation processes that are decisive for maintenance decisions.

This problem becomes especially important when condition assessment is extended towards RUL estimation. Classical diagnostics usually answers the question of how much damage is currently present in a belt section. In maintenance practice, however, additional questions are required: which transverse zones are degrading the fastest, when will they enter the refurbishment window, when will refurbishment cease to be economically justified, and when will the local condition become critical? Answering these questions requires not only a current health indicator, but also a prognostic framework that links degradation trajectories with remaining useful life estimation [1,4,5]. In maintenance decision-making, such prognostic information should then be connected with intervention thresholds, risk and cost considerations [17,18].

Several elements required for such a framework have already been developed in previous research, but they have usually been treated separately. Non-destructive testing methods for conveyor belts have been widely investigated and compared in terms of their principles, diagnostic capabilities, limitations, and suitability for industrial implementation. Sensor-based diagnostics has also been shown to support condition monitoring and predictive refurbishment of conveyor belts in industrial practice [2]. Consecutive DiagBelt scans have been used to identify damage development in steel-cord belt cores and to forecast remaining lifetime at the belt-section level using regression-based approaches [3,11]. In addition, the growth of longitudinal and transverse damage extents has been analysed as a time-dependent process [12], and transverse profiles of belt core damage have been shown to contain important information about loading quality and conveyor operation [16]. However, there is still a lack of an integrated approach that combines transverse, channel-resolved condition assessment with RUL estimation and a formal multi-threshold maintenance policy.

The limitation of average-based assessment is therefore not only a matter of data aggregation, but also a matter of decision quality. If a local critical zone forces a belt section to be removed from operation while the remaining part of the belt width still has usable residual life, the material potential of the belt is not fully utilized. This means that non-uniform cross-sectional degradation has both technical and economic consequences. It may increase the risk of unexpected failure, shorten the effective operating time of the belt section, reduce refurbishment potential, and increase the cost per unit operating time. Such a perspective is consistent with maintenance optimization research, in which technical condition, intervention timing, risk, and cost are treated as interconnected elements of maintenance decision-making [17,18].

The motivation of this paper is to move from average-based condition assessment towards sensor-based, channel-wise RUL estimation for steel-cord conveyor belts. The main idea is to represent the belt cross-section as a set of active diagnostic channels and to treat the local damage density in each channel as a health indicator evolving over time. Based on consecutive diagnostic scans, a simple accelerating degradation model is used to estimate the local degradation intensity and the remaining useful life of each channel. The resulting channel-wise RUL values are then linked with three decision thresholds corresponding to entry into the refurbishment window, the end of economically justified refurbishment, and the critical removal condition.

The main contributions of this paper are as follows:

- A channel-wise representation of belt cross-sectional condition is proposed, in which local damage density is treated as a spatially resolved health indicator derived from diagnostic scan data.
- A simple and interpretable accelerating degradation model is introduced for estimating local degradation intensity and channel-wise RUL.
- A three-threshold maintenance policy is formulated to distinguish between normal operation, the refurbishment window, operation after the refurbishment window, and the critical removal condition.
- Cross-sectional indicators are introduced to describe the fraction of channels exceeding each threshold, the width of compact critical zones, and the uniformity of life utilization across the belt width.
- A cost-oriented interpretation is proposed to show how non-uniform degradation may reduce effective belt life and increase the cost per unit operating time.

The proposed framework links sensor-based diagnostics, spatially resolved health indicators, RUL estimation, and maintenance decision support in a single structure. It provides a practical basis for condition-based maintenance and refurbishment planning of steel-cord conveyor belts, and it also creates a foundation for further integration with loading-condition analysis, transfer-chute assessment, and digital-twin-oriented conveyor diagnostics. This direction is consistent with recent attempts to use digital-twin concepts for conveyor-belt predictive maintenance, where sensor data, operating conditions and maintenance decision logic are combined in a virtual representation of the belt system [10,19].

## 2. Related Work and Research Gap

The literature relevant to this study can be grouped into five interconnected areas: sensor-based diagnostics and non-destructive testing of conveyor belts, health indicators and remaining useful life estimation, spatially resolved condition assessment in linear assets, mechanical causes of non-uniform belt degradation in loading zones, and maintenance decision-making based on multiple technical and economic thresholds. Together, these areas provide the methodological background for moving from average belt-section assessment towards channel-wise RUL estimation and multi-threshold maintenance support.

### 2.1. Sensor-Based Diagnostics and Non-Destructive Testing of Conveyor Belts

Conveyor belt diagnostics has developed from periodic visual inspection towards non-destructive, sensor-based and increasingly automated monitoring. Recent reviews show that a wide range of NDT techniques can be applied to conveyor belts, including magnetic flux leakage, X-ray imaging, ultrasonic testing, infrared thermography, machine vision, RFID-based methods and terahertz inspection [13]. The selection of a diagnostic method depends strongly on the type of belt, the type of expected damage, the required inspection speed and the need to detect either surface defects or hidden internal damage. In steel-cord belts, methods based on magnetic field disturbances are particularly important, because they enable the detection of hidden damage to the load-bearing steel-cord core without cutting the belt or interrupting its structure [13].

In parallel, vision-based and multi-sensor monitoring systems have been developed for real-time observation of conveyor belt operation. Chamorro et al. [20] demonstrated that machine vision combined with real-time sensor data can be used for conveyor belt health monitoring, while Guo et al. [21] proposed a lightweight vision-based damage detection approach suitable for industrial deployment. Recent deep-learning-based studies also show that conveyor belt damage detection is being developed for challenging mining conditions, including belt tear detection under practical visual disturbances [22].These studies confirm a broader trend in conveyor diagnostics: the diagnostic system is no longer treated only as an inspection device, but increasingly as a source of structured data for automated assessment and decision support. This trend is also visible in conveyor-system predictive maintenance, where sensor data are increasingly linked with decision algorithms, digital twins or data-driven models supporting maintenance planning rather than only alarm generation [10,23,24]. A similar data-driven direction is visible in recent conveyor-system studies, where machine-learning models are used to support fault detection and predictive maintenance of conveyor components [25].

However, most diagnostic approaches focus primarily on damage detection, classification, localization or current condition assessment. They provide information about whether damage exists, where it is located and how severe it is, but they do not always transform this information into a spatially resolved prognostic model. In particular, there is still a need to convert repeated diagnostic scans into local health indicators, degradation trajectories and maintenance-relevant RUL estimates. This is especially important for steel-cord conveyor belts, where the core damage detected by diagnostic systems may be spatially non-uniform across the belt width.

### 2.2. Health Indicators, Prognostics and RUL Estimation

In condition-based maintenance, diagnostics, prognostics and maintenance decision-making are usually treated as successive layers of one process. Jardine et al. [1] emphasized that machinery diagnostics and prognostics should support maintenance decisions by transforming condition monitoring data into information about current and future asset health. In this context, RUL estimation is one of the most important outputs of prognostics, because it expresses degradation in a time-oriented form that can be directly used for planning maintenance actions.

The RUL literature distinguishes several modelling families, including statistical data-driven models, physics-based models, hybrid models and machine-learning approaches [4,5]. Recent RUL studies also show that degradation may involve stochastic variability, random jumps, repair effects and parameter updates after preventive maintenance, which is important when interpreting simple degradation models as first-level approximations rather than complete physical descriptions [26,27]. Regardless of the modelling technique, a useful RUL framework typically requires three components: a health indicator that reflects the degradation state, a model describing the evolution of that indicator, and a failure or decision threshold that defines the end of acceptable operation [4,5]. This general structure is directly relevant to conveyor belts, because repeated diagnostic scans provide measurable damage indicators, while maintenance practice requires decisions about refurbishment, replacement or continued operation.

At the same time, many RUL studies are developed for single components or for systems represented by one dominant degradation signal. This is not fully sufficient for assets whose degradation has a spatial structure. In multi-sensor prognostics, recent approaches increasingly attempt to model not only temporal degradation, but also spatial or relational dependencies between sensor channels. Although the present paper does not use graph neural networks, this literature supports the general idea that RUL estimation may benefit from preserving relationships between local measurement channels rather than aggregating them into a single signal [28]. In steel-cord conveyor belts, damage may develop differently in individual transverse zones of the belt. Therefore, a single averaged health indicator may hide local zones with much shorter remaining life. The general RUL concept must therefore be adapted to the geometry and measurement structure of the belt, so that local diagnostic channels can be treated as separate health units.

### 2.3. Maintenance Thresholds and Decision-Oriented Prognostics

RUL estimation becomes practically useful only when it is connected with maintenance decisions. Maintenance optimization literature shows that intervention timing, inspection intervals, replacement decisions, cost, reliability and risk are strongly interdependent [17,18]. Therefore, maintenance policies should not be based solely on the prediction of failure time, but should also account for different operational states and different types of actions before failure occurs.

In many industrial systems, a single failure threshold is too simple to represent real maintenance practice. Threshold-based condition maintenance has a long tradition in reliability and maintenance optimization. Classical CBM models use alarm or intervention thresholds to trigger inspection, replacement or maintenance actions before failure occurs, while more recent work also considers dynamic thresholds and decision rules linked with RUL uncertainty [29,30]. Before an asset reaches a critical condition, it may first enter a warning zone, then a planning or repair window, and only later a state in which operation is no longer justified. This is particularly important for conveyor belts, because refurbishment has a limited technical and economic window. If the intervention is too early, part of the useful belt life is lost. If it is too late, refurbishment may become ineffective or economically unjustified. A multi-threshold policy is therefore more realistic than a single-threshold failure model, because it distinguishes between normal operation, the beginning of the refurbishment window, the end of economically justified refurbishment and the critical removal condition.

This decision-oriented interpretation is important for the present study. The aim is not only to estimate when a belt channel will reach a critical state, but also to determine when different maintenance-relevant thresholds will be crossed. In this way, RUL becomes a family of decision times rather than a single time-to-failure value.

### 2.4. Spatially Resolved Condition Assessment in Linear and Infrastructure Assets

The need for spatially resolved health indicators is not unique to conveyor belts. Similar problems occur in other linear or surface-like assets, such as pavements, railway tracks and bridges. Railway track deterioration models provide a particularly useful analogy, because track condition is commonly analysed in relation to spatial segments, deterioration mechanisms and whole-life asset management rather than as one global condition value for the entire system [31]. In pavement management, condition data are collected and interpreted at segment or profile level, because global condition indicators may not adequately represent local rutting, cracking or surface distress [32]. In bridge engineering, damage detection and condition rating methods increasingly consider the location, extent and severity of defects rather than only a single global condition score [33,34]. These examples show that when degradation is spatially heterogeneous, maintenance decisions should be supported by local indicators and spatial aggregation rules.

This methodological analogy is relevant to steel-cord conveyor belts. A belt section is a linear asset, but its load-bearing core also has a transverse structure. Local damage concentration in a selected part of the belt width may be much more important for future operation than the average damage density calculated for the whole section. Therefore, the diagnostic channel can be interpreted as a local condition unit, similar to a road segment, bridge component or localized damage zone in structural health monitoring. This analogy supports the transition from average belt-section indicators to channel-wise health indicators and spatially resolved RUL estimation.

### 2.5. Mechanical Causes of Non-Uniform Belt Degradation in Loading Zones

The spatial non-uniformity of belt damage is not only a diagnostic observation; it also has mechanical causes. Independent studies on conveyor belt damage mechanisms indicate that belt failures and local damage may result from falling material, impact loading, abrasive wear, belt mistracking and other operating factors, which supports the need to interpret transverse damage profiles in relation to conveyor operation rather than only as isolated defect maps [35,36]. In transfer and loading zones, the geometry of the chute, the trajectory of the material stream, impact velocity, shear interaction, abrasive work and off-centre loading may strongly influence the distribution of forces acting on the belt. The literature on transfer chute design and DEM modelling shows that the flow of bulk material in transfer points can be analysed and optimized to reduce impact, dust, noise and belt wear [37,38,39].

Kessler and Prenner [37] showed that DEM simulations can be used to analyse conveyor transfer chutes and support chute design. Doroszuk and Król [38] linked conveyor belt wear with material acceleration in transfer stations and emphasized the importance of feeding material onto the receiving belt with an appropriate velocity and direction. Ilic et al. [39] discussed bulk solid flow interactions in transfer chutes and showed that DEM and continuum approaches can support the analysis of accelerated and redirected material streams. These studies provide an important mechanical background for interpreting transverse belt damage profiles: if the material stream persistently acts more strongly on one part of the belt width, the diagnostic damage profile should also become spatially non-uniform.

In the present paper, DEM is not used as a modelling tool. Its role is interpretative. Transfer chute and DEM studies explain why localized belt degradation may occur, while diagnostic scans provide the measured effect of this non-uniform loading. In future work, channel-wise degradation coefficients obtained from diagnostic data may become a useful validation layer for mechanical models of loading zones and digital-twin-oriented conveyor diagnostics.

### 2.6. Conveyor Belt Damage Development and the Remaining Research Gap

Previous conveyor belt studies have already provided several elements needed for prognostic belt assessment. Consecutive diagnostic scans have been used to identify damage development in steel-cord belt cores and to forecast remaining lifetime at the belt-section level [3,11]. Transverse profiles of belt core damage have also been shown to contain information about loading quality and conveyor operation [16]. These works confirm that diagnostic data can be used not only for current condition assessment, but also for analysing degradation trends and interpreting operating conditions.

Nevertheless, these elements have not yet been fully integrated into one decision-oriented framework. Existing studies usually address one of the following aspects: detection of belt damage, monitoring of damage development, estimation of remaining lifetime at a more aggregated level, interpretation of transverse profiles, or optimization of maintenance decisions. What is still missing is a formal approach in which the belt cross-section is represented as a set of diagnostic channels, local damage density is treated as a channel-wise health indicator, RUL is estimated separately for each channel, and the resulting local life estimates are linked with a multi-threshold maintenance policy. To avoid ambiguity regarding the relationship between the present study and previous publications by the authors, Table 1 explicitly summarizes the differences between the present contribution and the most closely related earlier works. The novelty of the present paper is not the detection of belt damage itself, nor the use of repeated diagnostic scans alone, but the transformation of channel-resolved diagnostic data into local health indicators, channel-wise degradation trajectories, threshold-specific remaining useful life estimates, compact-zone indicators, and a multi-threshold maintenance policy.

This gap defines the contribution of the present paper. The proposed framework combines sensor-based diagnostic data, channel-wise health indicators, local degradation modelling, RUL estimation, compact critical-zone indicators and economic interpretation of non-uniform life utilization. In this way, it extends conveyor belt diagnostics from average-based condition assessment towards spatially resolved prognostic decision support.

## 3. Materials and Methods

**Figure 1:**
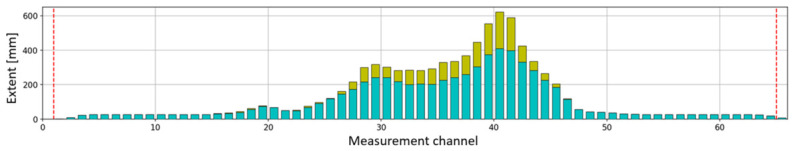
Non-uniform transverse distribution of steel-cord core damage in a conveyor belt section. Cyan bars indicate damage signals, yellow bars indicate accompanying signals, and red dashed vertical lines mark the belt width edges.

In practical conveyor belt maintenance, decisions concerning continued operation, refurbishment or replacement are often supported by aggregated condition indicators. A typical example is the average number of steel-cord core defects per unit belt length calculated for an entire belt section. Such indicators are simple, easy to communicate and useful for general classification of belt condition. However, they implicitly assume that the average condition of the section is sufficient to represent the degradation state relevant to maintenance decisions. This assumption may be misleading when belt degradation is spatially non-uniform. An example of a non-uniform transverse distribution of steel-cord core damage is shown in Figure 1.

A steel-cord conveyor belt is a linear asset with a load-bearing structure distributed across its width. During operation, individual belt sections and splices pass repeatedly through the same loading zones, but the mechanical interaction between the material stream and the belt is not necessarily uniform across the transverse direction. Chute geometry, off-centre loading, local impact of large particles, differences in material acceleration, and local cover wear may cause some parts of the belt cross-section to be exposed to more severe conditions than others [37,38,39]. Consequently, the steel-cord core may develop local zones of accelerated damage growth. Such zones may be narrow compared with the total belt width, but they can be decisive for the future usability of the entire belt section.

**Figure 2:**
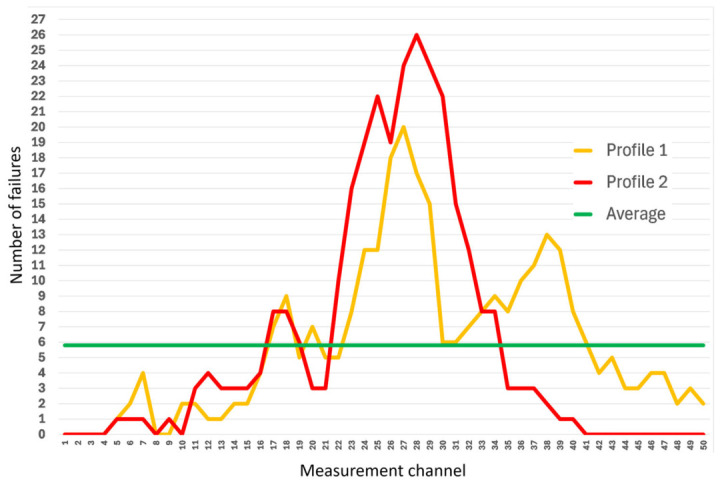
Schematic comparison of two transverse damage profiles with the same average damage density but different spatial distributions.

This creates a fundamental limitation of average-based assessment. Two belt sections may have the same average damage density, while their transverse damage profiles are completely different. In the first case, damage may be distributed relatively uniformly across the belt width. In the second case, most damage may be concentrated in a compact group of neighbouring channels, as schematically illustrated in Figure 2. From the point of view of maintenance, these two cases are not equivalent. Uniform degradation indicates that the belt width is being used relatively evenly, whereas localized degradation suggests the existence of a weak transverse zone that may enter a critical condition much earlier than the rest of the cross-section.

The problem is therefore not only diagnostic, but also prognostic. If the degradation process is spatially non-uniform, the remaining useful life of the belt section is not governed only by the average trajectory of damage growth. It may instead be governed by the local trajectory of the most degraded channel or compact group of channels. In such a case, the average indicator may overestimate the useful life of the belt section because it masks the local zone that will reach a decision threshold first. This issue is consistent with the general principles of prognostics, where an RUL model should be based on a health indicator that is sufficiently sensitive to the degradation mode relevant to the decision being made [1,4,5].

For steel-cord conveyor belts, the diagnostic data structure makes it possible to address this problem more explicitly. Non-destructive diagnostic scans provide information about the location of core damage along the belt and across its width. Previous studies have shown that consecutive scans can be used to analyse damage growth over time and estimate remaining lifetime at the belt-section level [3]. Other work has demonstrated that transverse profiles of belt core damage contain information about loading quality and conveyor operation [16]. These findings indicate that the transverse structure of damage should not be treated only as a visualization layer. It can be used as a basis for local health indicators and spatially resolved prognostic modelling.

**Figure 3:**
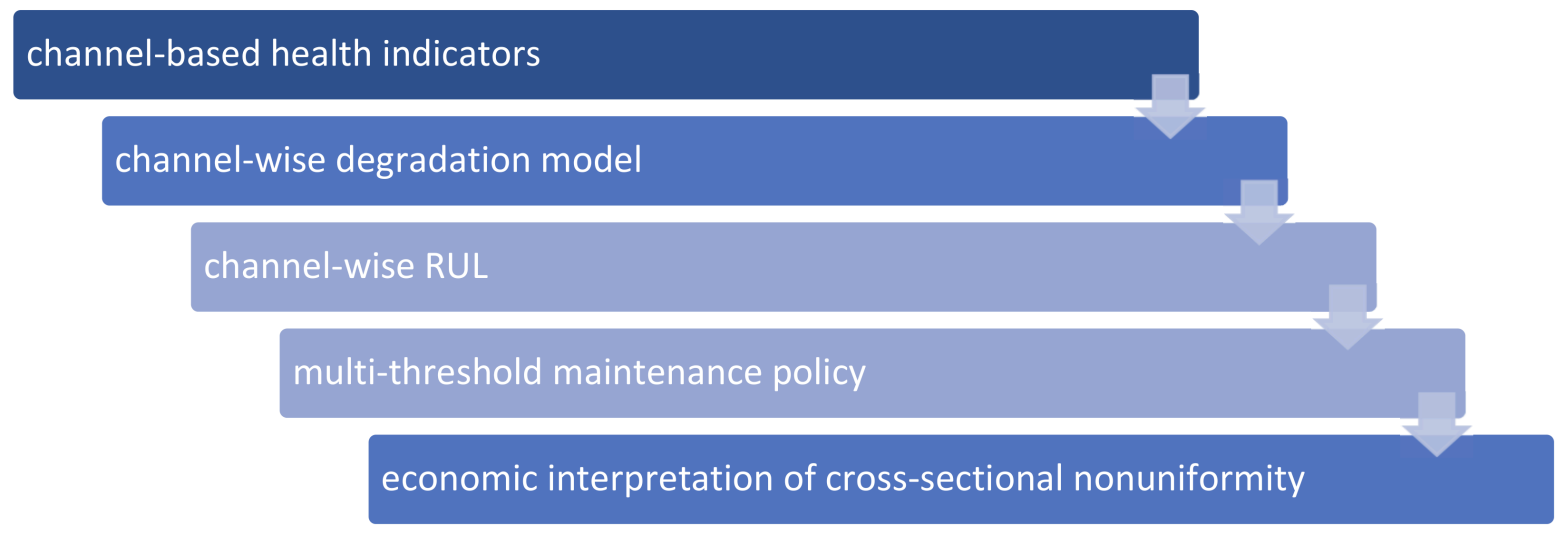
General framework for transforming channel-resolved diagnostic scan data into local degradation trajectories, channel-wise RUL estimates and maintenance decision indicators.

The central problem addressed in this paper can therefore be formulated as follows: how can diagnostic scan data be transformed from average belt-section indicators into channel-wise health indicators, channel-wise degradation trajectories and maintenance-relevant RUL estimates? The general logic of this transformation is presented in Figure 3. This question requires three methodological steps. First, the belt cross-section must be represented as a set of active diagnostic channels. Second, each channel must be assigned a local damage indicator that can be tracked over consecutive scans. Third, the local degradation trajectory must be linked with decision thresholds that correspond to practical maintenance actions.

The need for decision thresholds is particularly important. In real maintenance practice, a belt does not move directly from a fully acceptable condition to a failure state. Before critical removal becomes necessary, there is usually a period in which refurbishment is technically and economically justified. If refurbishment is planned too early, part of the useful belt life is lost. If it is planned too late, the belt may no longer be suitable for effective refurbishment or the expected benefits may not justify the intervention. This means that a single failure threshold is insufficient. A more realistic policy requires at least three decision levels: entry into the refurbishment window, the end of economically justified refurbishment, and the critical removal condition. Such multi-threshold logic is consistent with maintenance optimization research, where inspection, intervention timing, reliability and cost are treated as interconnected decision variables [17,18].

The motivation for the present study is also economic. If a local transverse zone reaches a critical state earlier than the rest of the belt width, the whole belt section may need to be removed from service even though other channels still have usable residual life. In this case, the material potential of the belt is not fully utilized. The cost of belt purchase, installation and maintenance is then distributed over a shorter effective operating time. Non-uniform cross-sectional degradation should therefore be interpreted not only as a technical problem, but also as a source of reduced life utilization and increased cost per unit operating time.

On this basis, the objective of the proposed framework is to shift the focus from the question “what is the average condition of the belt section?” to a more decision-oriented question: “which transverse zones determine the future life of the belt section, when will they cross maintenance thresholds, and how does their non-uniformity affect life utilization?” To answer this question, the following sections define channel-wise health indicators, introduce a local degradation model, formulate channel-wise RUL estimates for multiple thresholds, and propose cross-sectional indicators supporting maintenance decisions.

### 3.1. Diagnostic Data Structure and Channel-Wise Health Indicators

The objective of the proposed framework is to transform diagnostic scan data into spatially resolved health indicators that can later be used for degradation modelling and remaining useful life estimation. Instead of describing a belt section only by one average damage value, the belt cross-section is represented as a set of active diagnostic channels. Each channel corresponds to a local transverse zone of the belt and provides a separate unit for condition assessment.

**Figure 4:**
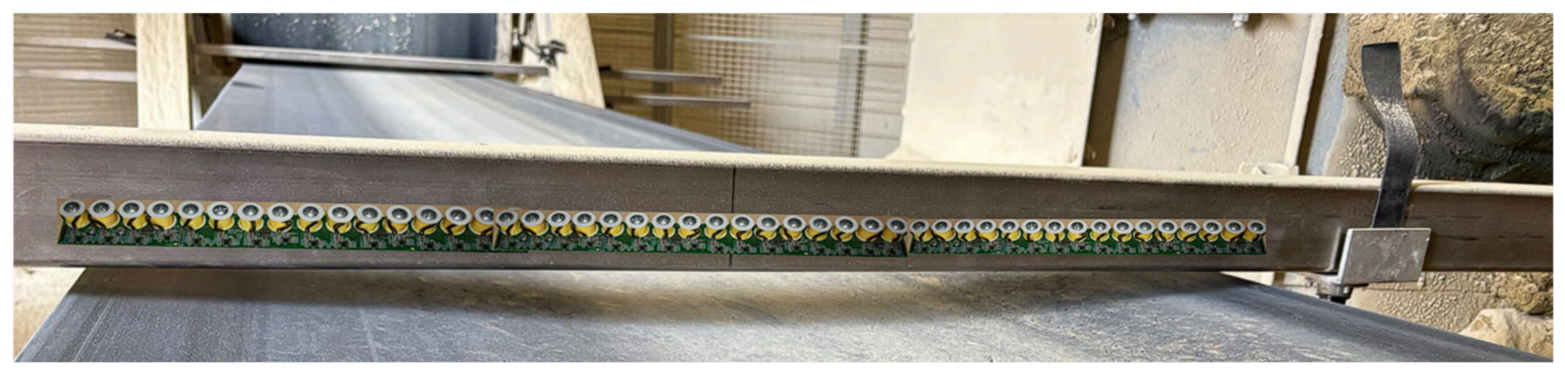
Measuring strip with diagnostic coils arranged across the belt width, defining active transverse measurement channels.

Let a belt section of length (L) be inspected during consecutive diagnostic campaigns indexed by (i = 1, 2, …, n), performed at times ($t_{i}$). The useful belt width is divided into (M) active measurement channels indexed by (k = 1, 2, …, M). The number of active channels depends on the width of the belt, the geometry of the diagnostic sensor array and the part of the cross-section effectively covered by measurement. In the diagnostic system considered in this study, the transverse measurement structure is related to the arrangement of coils installed in the measuring head, with a spacing of 25 mm between neighbouring channels. The general idea of the measuring strip and channel arrangement is shown in Figure 4.

To clarify the formation of the regression dataset, the spatial aggregation procedure is defined explicitly. In this study, a belt section is understood as an analysed longitudinal fragment of the belt with length L, while a scan denotes one diagnostic inspection campaign performed at time $t_{i}$. During each scan, steel-cord core defects are detected along the belt length and assigned to transverse diagnostic channels according to their lateral position in the measuring head.

For a given scan time $t_{i}$ and channel *k*, all defects assigned to this channel within the analysed belt section are aggregated along the entire section length L. The resulting value $N_{k}(t_{i})$ is therefore the total number of defects detected in channel *k* over the analysed belt section during scan *i*. The corresponding channel damage density is then calculated as $d_{k}(t_{i})$. Thus, the regression observation is a section-level, channel-specific damage density. It is not a point measurement at a single longitudinal coordinate and it is not a transverse profile at one isolated belt cross-section. For each scan (i) and each channel (k), the diagnostic system identifies steel-cord core defects assigned to the corresponding transverse zone. The basic diagnostic variable is therefore the number of defects observed in channel (k) during scan (i), denoted as:

$$N_{k}(t_{i})$$

where $N_{k}(t_{i})$ is the defect count assigned to channel (k) at time ($t_{i})$. This count-based representation is consistent with many practical diagnostic procedures, where the number of detected core defects is used as a primary indicator of belt condition [3,11,13]. However, because belt sections may have different lengths, the raw number of defects should be normalized by the analysed section length. For this reason, the local channel damage density is defined as:

$$d_{k}(t_{i})=\frac{N_{k}(t_{i})}{L}$$

where $d_{k}(t_{i})$ is the count-based local health indicator expressed as the number of defects per metre of belt section.

If the diagnostic system also provides information about the surface area or size of defects, the same framework can be extended to area-based indicators. Let $A_{k}(t_{i})$ denote the total damaged area assigned to channel (k) during scan (i). The local area-based damage density can then be defined as:

$$a_{k}\left(t_{i}\right)=\frac{A_{k}(t_{i})}{L}$$

where $a_{k}\left(t_{i}\right)$ represents the damaged area per metre of belt section. In the present study, the main formulation is developed for the count-based indicator $d_{k}(t_{i})$, because it is simple, robust and directly related to the diagnostic data structure used in industrial inspections. Nevertheless, all subsequent steps can be adapted to area-based or combined indicators if such information is available.

For a given scan, the vector of channel-wise damage densities is defined as:

$$d\left(t_{i}\right)=\left[d_{1}\left(t_{i}\right),\;d_{2}\left(t_{i}\right),\ldots ,d_{M}(t_{i})\right]$$

This vector describes the transverse damage profile of the belt section. It preserves information about the spatial distribution of degradation across the belt width. This is important because previous studies have shown that transverse damage profiles may contain information about loading quality and conveyor operation [16]. Therefore, the profile $d\left(t_{i}\right)$ should not be treated only as a visualization of defects, but as a set of local health indicators.

The classical average damage density for the whole belt cross-section is obtained as:

$$\bar{d}\left(t_{i}\right)=\frac{1}{M}\sum_{k=1}^{M}d_{k}(t_{i})$$

This value is useful because it remains compatible with traditional section-level condition assessment. However, it is only one aggregation of the full channel-wise profile. It does not describe whether damage is uniformly distributed or concentrated in a narrow part of the belt width. Therefore, the proposed framework also uses additional spatial indicators.

The maximum local damage density is defined as:

$$d_{max}\left(t_{i}\right)=\underset{1\leq k\leq M}{\max}d_{k}(t_{i})$$

This indicator identifies the most degraded channel in the belt cross-section. To quantify the relation between the most degraded local zone and the average state of the section, the damage concentration coefficient is introduced:

$$C_{d}\left(t_{i}\right)=\frac{d_{max}(t_{i})}{\bar{d}(t_{i})}$$

High values of $C_{d}\left(t_{i}\right)$ indicate that the belt section contains a local damage peak that is much more severe than the average cross-sectional condition. In such a case, the average indicator may underestimate the importance of localized degradation.

To describe the overall non-uniformity of the transverse damage profile, the standard deviation of channel-wise damage density is calculated as:

$$\sigma_{d}\left(t_{i}\right)=\;\sqrt{\frac{1}{M}\sum_{k=1}^{M}{\left(d_{k}\left(t_{i}\right)-\bar{d}(t_{i})\right)}^{2}}$$

The coefficient of variation is defined as:

$$CV_{d}\left(t_{i}\right)=\frac{\sigma_{d}(t_{i})}{\bar{d}(t_{i})}$$

The coefficient $CV_{d}\left(t_{i}\right)$ is a dimensionless measure of cross-sectional non-uniformity. Low values indicate relatively uniform degradation across the belt width, whereas high values indicate strong local concentration of damage. Similar logic is used in other forms of condition monitoring and infrastructure assessment, where local or segment-level indicators are preferred when degradation has a spatial structure [1,5,32].

In maintenance practice, it is also important to determine whether highly degraded channels are isolated or whether they form a compact transverse zone. For a given threshold ($T_{j}$), the set of channels exceeding this threshold is defined as:

$$\Omega_{j}\left(t_{i}\right)=\;\left(k\in \left\{1,2,\ldots ,M\right\}:d_{k}(t_{i})\geq T_{j}\right)$$

The number of channels exceeding threshold ($T_{j}$), is:

$$m_{j}\left(t_{i}\right)=\;\Omega_{j}(t_{i})$$

The corresponding fraction of the belt width affected by degradation above this threshold is:

$$p_{j}\left(t_{i}\right)=\frac{m_{j}(t_{i})}{M}$$

where j denotes the threshold level. In the proposed framework, three thresholds are later used: entry into the refurbishment window, the end of economically justified refurbishment and the critical removal condition.

The spatial compactness of degradation is described by the width of the largest contiguous block of neighbouring channels exceeding threshold ($T_{j}$). This quantity is denoted as:

$$B_{j}(t_{i})$$

where $B_{j}(t_{i})$ is expressed as the number of adjacent channels. If the physical spacing between channels is known, $B_{j}(t_{i})$ can also be converted into a physical width. This indicator is important because a compact group of highly degraded neighbouring channels may have greater operational significance than the same number of isolated channels distributed across the belt width.

The main variables and indicators used in the proposed framework are summarized in Table 2.

### 3.2. Channel-Wise Degradation Model

The channel-wise health indicators defined in the previous section describe the current transverse condition of a belt section. However, maintenance decisions require not only information about the present state, but also information about the future development of degradation. For this reason, the next step of the proposed framework is to model the evolution of local damage density in each diagnostic channel over time. It should be emphasized that fitting a separate degradation trajectory to each channel is a modelling and computational assumption, not an assumption of full mechanical independence between neighbouring transverse zones. In real conveyor operation, adjacent channels may be mechanically correlated because they are affected by the same material stream, loading zone, belt mistracking, impact conditions and local cover wear. The channel-wise model is therefore used to preserve local diagnostic information, while spatial dependence is partly reflected later by compact-zone indicators describing contiguous groups of neighbouring channels exceeding the same threshold.

**Figure 5:**
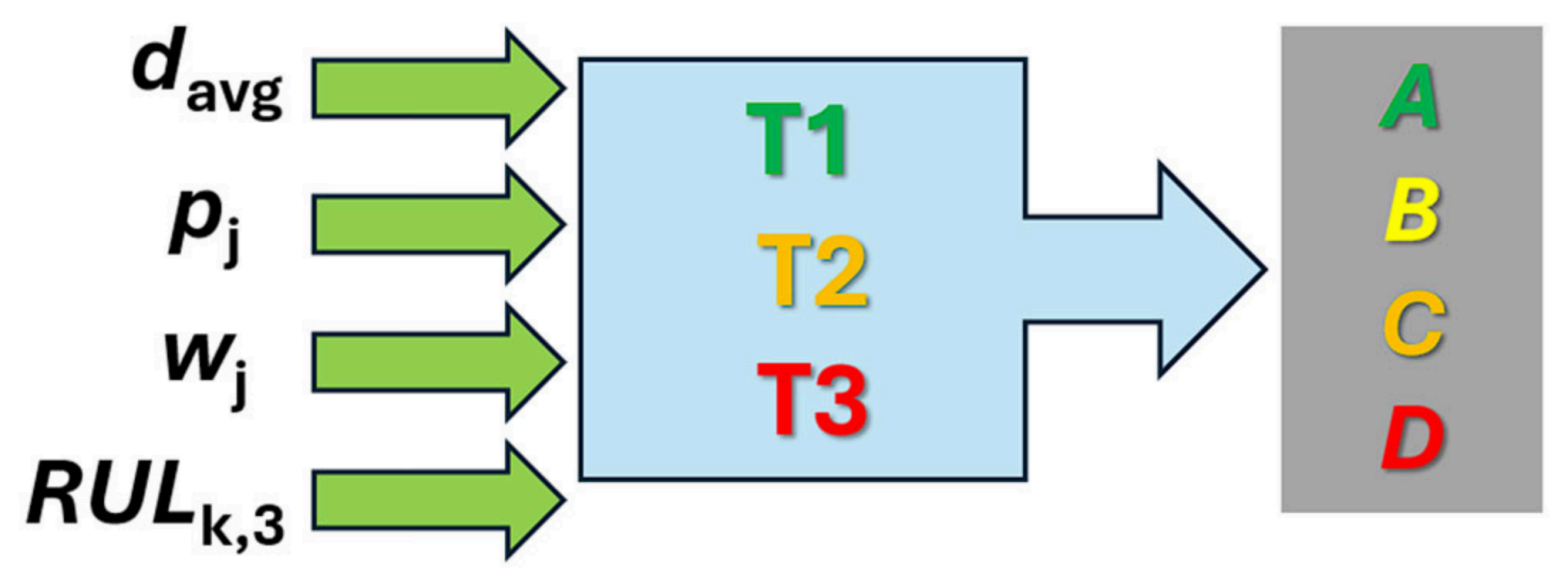
Conceptual workflow linking channel-wise diagnostic indicators, threshold levels and maintenance states.

Let consecutive diagnostic scans be performed at times $t_{1},\;t_{2},\;\ldots ,\;t_{n}$. For each channel k, the diagnostic data form a time series:

$$d_{k}(t_{1}),\;d_{k}(t_{2}),\;\ldots ,\;d_{k}(t_{n})$$

where $d_{k}(t_{i})$ is the local damage density in channel *k* at scan time $T_{j}$. In this formulation, each channel is treated as a local degradation unit. This means that one belt section is not represented by a single degradation trajectory, but by a set of M local trajectories corresponding to M active diagnostic channels. The general workflow from diagnostic scans to channel-wise health indicators, local degradation trajectories and RUL estimation is shown in Figure 5.

The proposed model is based on the assumption that local damage density increases with time according to a simple accelerating degradation law:

$$d_{k}(t)\;=\;\alpha_{k}\;\cdot \;t^{2}$$

where $\alpha_{k}$ is the local degradation acceleration coefficient for channel k. The coefficient $\alpha_{k}$ describes the intensity of damage growth in a given transverse zone of the belt. Higher values of $\alpha_{k}$ indicate faster local degradation and, consequently, shorter time to reach maintenance thresholds.

The choice of this model is intentional. In prognostics and condition-based maintenance, degradation models are commonly used to transform condition-monitoring data into estimates of future state and remaining useful life [1,4,5]. Statistical and data-driven RUL approaches generally require three elements: a measurable health indicator, a model describing the evolution of this indicator, and a threshold defining the end of acceptable operation or the need for intervention [4,5,41,42]. In the present study, this general structure is adapted to channel-resolved conveyor belt diagnostics.

The quadratic form is used as a first-level approximation of accelerating degradation. This choice is consistent with the general use of parametric degradation models in RUL estimation, where relatively simple mathematical functions are often preferred when the number of observations is limited and interpretability is required for maintenance decision support [4,41,42]. The objective is not to construct the most complex degradation model, but to obtain a transparent relationship between measured damage density, local degradation intensity and time to maintenance thresholds.

The quadratic model should therefore be interpreted as a first-level, interpretable approximation of accelerating damage growth rather than as a universally superior degradation law. The present industrial case study is based on four diagnostic scans, which limits the reliability of formal model selection between linear, quadratic, exponential, power-law or piecewise linear alternatives. With such a sparse diagnostic history, fitting several alternative nonlinear models may lead to unstable parameter estimates and overinterpretation of goodness-of-fit measures. Therefore, the adopted model is used to demonstrate the proposed channel-wise RUL framework, while future applications based on longer diagnostic histories should compare candidate degradation models using RMSE, MAE, R^2^, AIC/BIC or predictive error on deferred scans.

The physical interpretation of the accelerating form is also consistent with belt operation. As the belt cover becomes locally worn and the steel-cord core becomes increasingly exposed or weakened, the probability of further damage in the same transverse zone may increase. Therefore, a channel with already intensified degradation may continue to degrade faster than less affected channels. The model does not attempt to describe every individual damage event. Instead, it approximates the cumulative effect of many operating cycles, impacts and local loading conditions on the observed damage density.

For steel-cord conveyor belts, consecutive diagnostic scans provide a practical basis for estimating degradation trends because they allow the development of core damage to be observed over time [3,11]. In contrast to earlier belt-section-level approaches, the present framework fits a separate degradation model to each active diagnostic channel. As a result, the degradation process is represented not by one average curve, but by a transverse set of local degradation trajectories.

For each channel k, the coefficient $\alpha_{k}$ can be estimated from consecutive diagnostic scans. If time is measured from a common reference point, such as belt installation or the first diagnostic scan, the least-squares estimate of $\alpha_{k}$ for the model without intercept can be written as:

$$\alpha_{k}=\frac{\sum_{i=1}^{n}t_{i}^{2}\cdot d_{k}(t_{i})}{\sum_{i=1}^{n}t_{i}^{4}}$$

This formula provides one degradation acceleration coefficient for each diagnostic channel. The complete transverse profile of degradation acceleration is therefore:

$$\alpha =[\alpha_{1},\;\alpha_{2},\;\ldots ,\;\alpha_{M}]$$

This profile is one of the key outputs of the proposed framework. While $d_{k}(t_{i})$ describes the current local condition, $\alpha_{k}$ describes how fast the condition of channel k is deteriorating. Therefore, two channels may have similar current damage densities but different future behaviour if their $\alpha_{k}$ values are different. A channel with a higher $\alpha_{k}$ value will reach the same decision threshold earlier than a channel with a lower $\alpha_{k}$ value.

For comparison with the classical average-based approach, an analogous model can be fitted to the average cross-sectional damage density:

$$\bar{d}\left(t\right)=\bar{\alpha }\cdot t^{2}$$

where $\bar{d}\left(t\right)$ is the average damage density of the belt cross-section and $\bar{\alpha }$ is the degradation acceleration coefficient estimated for the average profile. This average model is useful as a reference, but it does not preserve information about local differences between channels. If the degradation profile is nearly uniform, the average model may provide a reasonable approximation of the section condition. However, if the degradation profile is strongly non-uniform, the average model may mask the most critical local trajectories.

The quadratic model should therefore be understood as a first-level interpretable approximation suitable for sparse diagnostic histories. With longer time series and additional operating data, the framework could be extended using stochastic degradation models, Bayesian updating, state-space formulations, jump-diffusion processes or particle filtering methods [26,27,41].

The distinction between $\bar{\alpha }$ and $\alpha_{k}$ is central to the proposed approach. The average coefficient $\bar{\alpha }$ represents the general degradation tendency of the whole belt section. In contrast, each local coefficient $\alpha_{k}$ represents the degradation tendency of one transverse zone. Channels with high $\alpha_{k}$ values can be interpreted as zones of accelerated life consumption. These zones may be associated with persistent local loading, asymmetric material flow, impact concentration or local weakening of the belt structure. In this sense, the profile of $\alpha_{k}$ values is not only a mathematical output of regression, but also a diagnostic signature of non-uniform belt operation.

The model should be understood as a first-level prognostic model. It does not explicitly include sudden damage events, changes in loading conditions, belt repairs, splice replacement or operational disturbances between scans. It also assumes that the degradation trend observed in previous scans remains representative for the near future. These assumptions are simplifications, but they make the model transparent and suitable for maintenance decision support. More complex models, including probabilistic, Bayesian, stochastic-process-based or load-dependent formulations, can be introduced in future work when longer diagnostic histories and operating data are available [4,41,42].

The output of this section is therefore a set of channel-wise degradation trajectories and a transverse profile of local degradation acceleration coefficients. These outputs provide the direct input for the next step of the framework: estimating the time remaining until each channel reaches defined maintenance thresholds.

### 3.3. Channel-Wise RUL Estimation and Multi-Threshold Maintenance Policy

The degradation model defined in the previous section provides a local trajectory of damage density for each diagnostic channel. The next step is to translate these trajectories into remaining useful life values and maintenance decisions. In the proposed framework, RUL is not defined only as the time remaining to a final critical state. Instead, it is calculated with respect to several decision thresholds corresponding to different maintenance actions.

For channel k, the remaining useful life is defined as the time from the most recent diagnostic scan to the moment when the local damage density reaches a specified threshold $T_{j}$. For the accelerating degradation model:

$$d_{k}\left(t\right)=\alpha_{k}t^{2}$$

The time at which channel k reaches threshold $T_{j}$ is:

$$t_{k,j}=\sqrt{\frac{T_{j}}{\alpha_{k}}}$$

where $T_{j}$ is the j-th decision threshold and $\alpha_{k}$ is the degradation acceleration coefficient of channel k. If the most recent diagnostic scan was performed at time $t_{n}$, the channel-wise remaining useful life is:

$$RUL_{k,j}\left(t_{n}\right)=t_{k,j}-t_{n}$$

or, equivalently:

$$RUL_{k,j}\left(t_{n}\right)=\sqrt{\frac{T_{j}}{\alpha_{k}}}-t_{n}$$

This value represents the estimated time remaining until channel k reaches threshold $T_{j}$. If $RUL_{k,j}\left(t_{n}\right)$ is positive, the threshold has not yet been reached. If it is equal to zero or negative, the threshold has already been exceeded.

Three thresholds are introduced in this study:

- $T_{1}$—entry into the refurbishment window;
- $T_{2}$—end of economically justified refurbishment;
- $T_{3}$—critical removal condition.

The numerical values of thresholds T1–T3 should be treated as decision-policy parameters rather than universal material constants. In practice, their determination should be based on a combination of historical diagnostic records, previous refurbishment and replacement decisions, post-removal belt condition assessment, laboratory strength testing of belt samples with different damage levels, refurbishment feasibility limits, and the risk level accepted by the belt user. Therefore, the proposed framework does not prescribe one universal set of threshold values, but provides a procedure in which threshold levels can be calibrated for a specific belt type, diagnostic method, operating environment and maintenance strategy. This interpretation is consistent with recent RUL studies in which maintenance thresholds are treated as decision parameters that may depend on degradation uncertainty, maintenance influence and operational policy.

The sensitivity of the decision time to threshold selection follows directly from the adopted degradation model. For the quadratic degradation law, the estimated time to threshold is proportional to the square root of the threshold value. Therefore, lowering a threshold leads to earlier predicted intervention, while increasing a threshold delays the predicted decision time. A conservative policy can be represented by lower values of T1–T3, whereas a more permissive policy shifts the same decision states to later operating times but increases operational risk. For this reason, threshold calibration should be accompanied by sensitivity analysis in which conservative, baseline and permissive threshold scenarios are compared (Table 3).

Thus, for each channel k, three RUL values can be calculated:

- $RUL_{k,1}$—time remaining to enter the refurbishment window;
- $RUL_{k,2}$—time remaining to the end of economically justified refurbishment;
- $RUL_{k,3}$—time remaining to the critical removal condition.

The same calculation can be performed for the average cross-sectional damage density. If the average degradation model is:

$$\bar{d}\left(t\right)=\;\bar{\alpha }t^{2}$$

then the average-based RUL for threshold $T_{j}$ is:

$$RUL_{avg,j}\left(t_{n}\right)=\;\sqrt{\frac{T_{j}}{\bar{\alpha }}}-t_{n}$$

The comparison between $RUL_{avg,j}$ and $RUL_{k,j}$ is important for interpreting non-uniform cross-sectional degradation. If degradation is approximately uniform across the belt width, local RUL values should be close to the average-based RUL. If degradation is strongly non-uniform, selected channels may reach the same thresholds much earlier than the average profile suggests.

**Figure 6:**
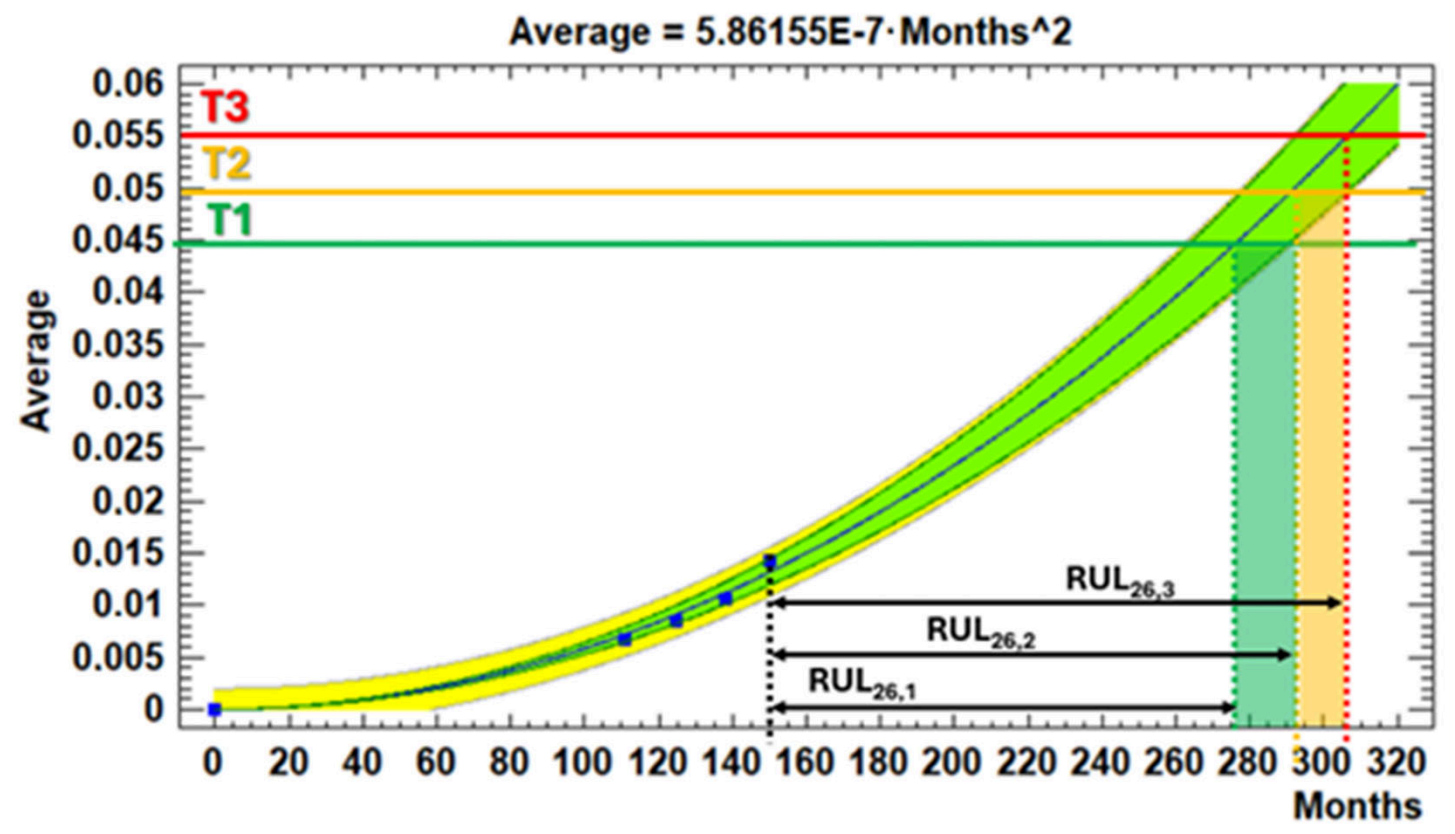
Channel degradation trajectory and threshold-based RUL definition for average channel density.

**Figure 7:**
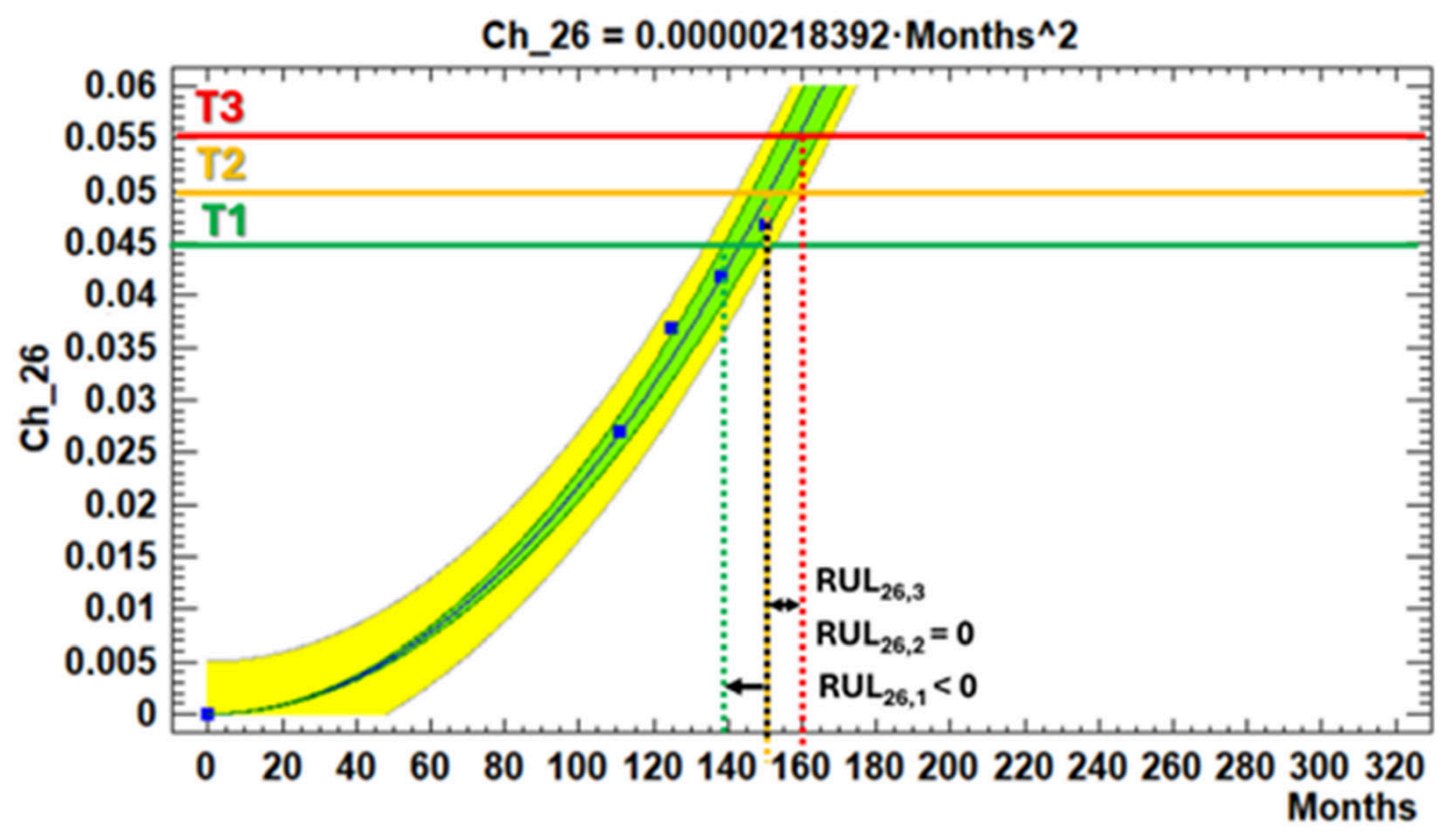
Channel degradation trajectory and threshold-based RUL definition for selected channel 26.

Figure 6 presents the threshold-based RUL definition for the average damage density. Figure 7 presents the same logic for a selected local channel. In the analysed example, the selected channel has already exceeded two lower thresholds, and only the RUL to the critical removal condition remains positive. This illustrates the main limitation of average-based assessment: the average trajectory may still indicate a moderate condition, while a local channel has already entered a much more advanced degradation state.

The length of the refurbishment window for channel k is defined as the difference between the time to threshold $T_{2}$ and the time to threshold $T_{1}$:

$$RW_{k}=RUL_{k,2}-RUL_{k,1}$$

This value represents the estimated period in which refurbishment remains technically and economically justified for channel k. Channels with high degradation acceleration have shorter refurbishment windows, which reduces the time available for planning maintenance actions.

To describe the life of the belt section as a whole, the minimum local RUL is introduced:

$$RUL_{min,j}\left(t_{n}\right)=\underset{1\leq k\leq M}{\min}RUL_{k,j}(t_{n})$$

This indicator identifies the most critical transverse zone of the belt. In a non-uniformly degraded belt section, $RUL_{min,j}$ may be much shorter than $RUL_{avg,j}$. Therefore, the practical life of the section may be governed by the local minimum rather than by the average condition.

A simple life-uniformity indicator can be defined as:

$$U_{j}\left(t_{n}\right)=\frac{RUL_{min,j}(t_{n})}{RUL_{avg,j}(t_{n})}$$

Values of $U_{j}$ close to 1 indicate that the cross-section degrades relatively uniformly. Low values indicate that the life of the entire belt section is limited by one local zone or a compact group of channels with much shorter remaining life.

The proposed maintenance policy combines threshold crossing, local RUL and cross-sectional extent of degradation. Four operational states are distinguished (Table 4):

Such a multi-state interpretation follows the general logic of CBM policies, in which the observed degradation state is mapped into maintenance actions through warning, intervention or replacement thresholds rather than through a single failure boundary [17,29,30].

A belt section is classified into these states using both average and channel-wise indicators. The average condition provides a general view of the belt section, while channel-wise indicators reveal whether a local critical zone has already developed. The fraction of channels exceeding each threshold, the width of compact zones above threshold and the minimum local RUL are therefore used together with the average RUL.

The most important decision rule is that a belt section should not be considered safe only because its average condition remains below the critical threshold. If a compact group of channels exceeds $T_{3}$ or if $RUL_{min,3}$ is close to zero, the section should be treated as critical even if the average profile suggests a longer remaining life. This rule reflects the practical assumption that the future usability of a belt section may be limited by the weakest transverse zone.

**Figure 8:**
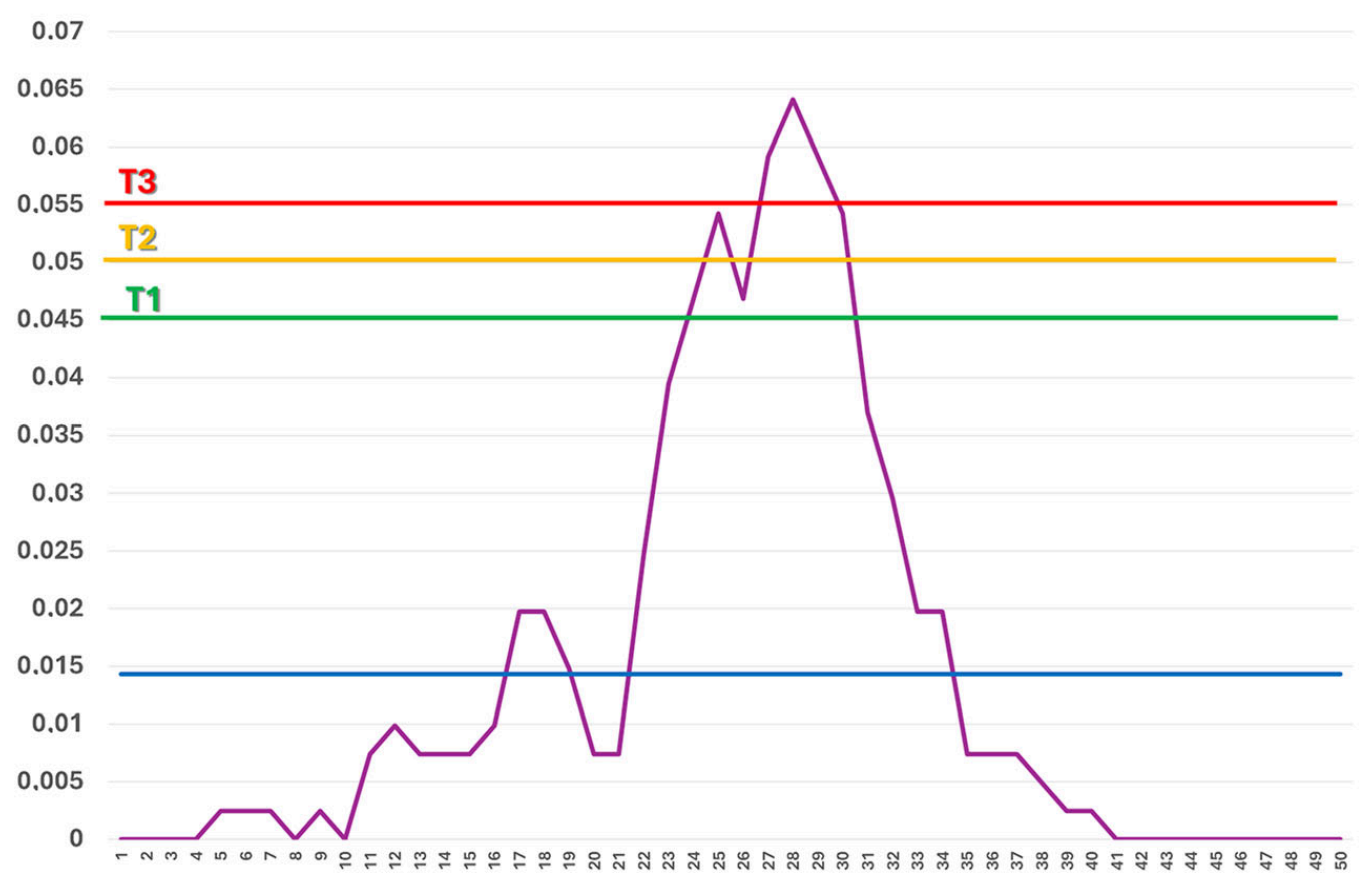
Example of threshold-based classification of a transverse damage profile. The blue line represents the mean damage density across all channels, while the purple curve shows the damage density for each measurement channel across the width of the belt.

The general decision logic of the three-threshold maintenance policy is shown in Figure 8.

The proposed policy transforms channel-wise diagnostic information into maintenance-relevant decisions. Instead of producing only one average RUL value, it identifies which channels enter the refurbishment window first, which channels leave this window, and which zones approach the critical removal condition. This provides a more realistic basis for condition-based maintenance, because it accounts for spatially non-uniform degradation across the belt width.

## 4. Results—Industrial Case Study

The proposed framework was applied to diagnostic data obtained from an industrial steel-cord conveyor belt loop operated in an underground coal mine. The analysed belt loop was inspected four times using the DiagBelt+ diagnostic system. The total operating time of the analysed belt section was approximately 150 months. The data used in this case study include channel-resolved information about steel-cord core damage obtained from consecutive diagnostic scans.

The detailed demonstration of the proposed framework was performed for an industrial steel-cord conveyor belt operated in an underground coal mine. The belt was inspected during four diagnostic campaigns covering approximately 150 months of operation. To improve the reproducibility of the case study and to define the scope of the analysed dataset, the main characteristics of the diagnostic data are summarized in Table 5.

Some operational details of the industrial conveyor system are not disclosed for confidentiality reasons. However, all parameters required to understand the diagnostic data structure, spatial aggregation procedure and regression dataset used in the proposed framework are provided.

The purpose of the case study is not to define universal threshold values for all conveyor belts, but to demonstrate how repeated diagnostic scans can be transformed into channel-wise degradation trajectories and how these trajectories differ from the average belt-section trend. Such an approach is consistent with the general direction of sensor-based condition monitoring, in which diagnostic systems are increasingly used not only for defect detection, but also for degradation tracking and maintenance decision support [1,13,20].

For each diagnostic scan, the number of detected core defects was assigned to transverse diagnostic channels. The local channel damage density was then calculated according to the definitions introduced in Section 4. In addition, the average damage density for the analysed section was calculated as a reference indicator. This made it possible to compare the classical average-based assessment with the proposed channel-wise approach.

**Figure 9:**
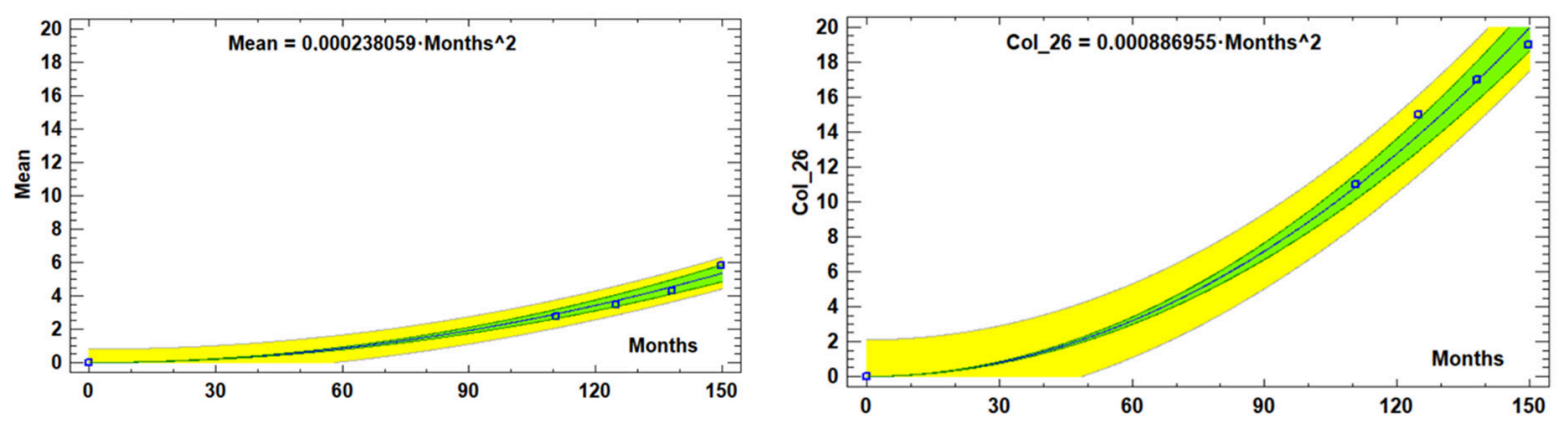
Regression curves of the number of defects for the average belt-section indicator and for the selected diagnostic channel 26.

Figure 9 presents regression curves fitted to the average damage count and to one selected diagnostic channel, channel 26. The selected channel corresponds to a transverse zone in which the development of damage was considerably faster than the average trend for the whole belt section.

The comparison shown in Figure 9 illustrates the main limitation of average-based assessment. The average damage curve increases relatively slowly because it represents the whole belt width, including areas where almost no defects were detected. In contrast, channel 26 shows a much faster increase in damage. This means that the average indicator smooths the transverse variability of degradation and may hide a local zone that is losing useful life more rapidly.

From the point of view of maintenance decision-making, this difference is essential. If RUL estimation were based only on the average curve, the analysed belt section would appear to have a longer remaining life. However, the channel-wise model indicates that at least one local transverse zone may reach decision thresholds significantly earlier. In practice, this means that the future usability of the entire belt section may be controlled by the most degraded channel or compact group of channels, rather than by the average condition of the belt width.

The case study also confirms the diagnostic importance of transverse damage profiles. In the analysed belt section, damage was not uniformly distributed across the belt width. A large part of the damage concentration occurred in selected central channels, while some edge zones showed little or no damage. Such a profile suggests that the belt cross-section was not used uniformly during operation. This observation is consistent with previous findings that transverse profiles of steel-cord core damage may reflect loading quality and conveyor operating conditions [3,16].

The practical value of the proposed approach is therefore twofold. First, it provides a more sensitive basis for RUL estimation, because local degradation trajectories can be analysed separately from the average trend. Second, it provides additional diagnostic information about the spatial organization of damage. Channels with high degradation acceleration coefficients can be treated as local indicators of accelerated life consumption and may point to persistent mechanical causes, such as asymmetric loading, local impact concentration or non-uniform material flow in the transfer zone.

This case study demonstrates that consecutive diagnostic scans can be used not only to describe the current condition of a belt section, but also to identify transverse zones that dominate future maintenance decisions. The results support the need for channel-wise RUL estimation and justify the use of multi-threshold maintenance policy introduced in the previous section.

## 5. Economic Interpretation

Non-uniform degradation of the belt cross-section has not only diagnostic and prognostic significance, but also economic consequences. If one local transverse zone reaches a critical condition much earlier than the rest of the belt width, the entire belt section may need to be removed from operation before the remaining channels have fully utilized their potential life. In this case, part of the material capacity of the belt is lost. The belt is not removed because the whole cross-section is uniformly worn, but because a local weak zone limits the usability of the section.

This effect can be interpreted as a reduction in life utilization efficiency. In a uniformly degraded belt section, local channel lives are similar, and the belt width is used relatively evenly. In a non-uniformly degraded section, the minimum local RUL may be much shorter than the average-based RUL. As a result, the effective operating time of the whole section is governed by the most critical channel or compact group of channels. This mechanism links cross-sectional damage concentration directly with maintenance cost.

Let $C_{tot}$ denote the total cost associated with purchasing, installing, operating and maintaining a belt section over its useful life. A simplified cost per unit operating time can be expressed as:

$$C_{time}=\frac{C_{tot}}{t_{use}}$$

where $t_{use}$ is the effective operating time of the belt section until refurbishment or removal. If non-uniform degradation forces earlier removal, $t_{use}$ decreases and $C_{time}$ increases. This simplified formulation is consistent with the general logic of maintenance optimization, in which intervention timing, asset condition and cost are treated as interconnected decision variables [1,17,18].

To express the utilization of the belt width life potential, a simple life utilization efficiency indicator can be introduced:

$$E_{life,j}=\frac{RUL_{min,j}}{RUL_{avg,j}}$$

where $RUL_{min,j}$ is the minimum local RUL to threshold $T_{j}$, and $RUL_{avg,j}$ is the RUL estimated from the average cross-sectional damage density. Values close to 1 indicate that the belt cross-section is used relatively uniformly. Low values indicate that the whole section is limited by a local zone with much shorter remaining life.

The economic interpretation is especially important for refurbishment planning. If a belt section enters the refurbishment window uniformly, the decision can be planned based on the overall section condition. If only a narrow local zone enters or leaves this window much earlier than the average profile, the decision becomes more difficult. Refurbishment may be technically possible for most of the belt width, but economically or mechanically limited by the most damaged channels. This shows why the proposed framework distinguishes between average RUL, minimum local RUL, the fraction of channels above each threshold and the width of compact critical zones.

The proposed cost-oriented indicators should be treated as a first-level interpretation rather than a complete economic model. They do not include discounting, uncertainty of failure between scans, downtime cost, production losses, spare belt availability or alternative maintenance schedules. However, they show that cross-sectional non-uniformity is a measurable source of economic inefficiency. Reducing local damage concentration by improving loading conditions, chute geometry or material flow distribution may therefore increase not only technical safety, but also the effective economic use of the belt.

In this sense, channel-wise diagnostics can support two types of decisions. First, it can support direct belt maintenance decisions, such as refurbishment or replacement planning. Second, it can provide feedback on the quality of conveyor operation, especially in loading zones. A persistent concentration of degradation in selected channels may indicate that the belt is being used unevenly, which can justify corrective actions in the transfer point. The economic benefit of such actions should be evaluated by comparing their cost with the expected extension of belt life and reduction in premature belt removal.

## 6. Discussion

The proposed framework shows that the condition and remaining useful life of a steel-cord conveyor belt section should not be interpreted only through average damage indicators. Average values are useful for general condition classification, but they may hide local transverse zones in which degradation progresses much faster. This is particularly important for RUL estimation, because the practical life of a belt section may be governed by the most degraded channel or compact group of channels rather than by the average cross-sectional condition. This observation is consistent with the general principles of prognostics, where the selected health indicator should be sensitive to the degradation mode that determines the maintenance decision [1,4,5].

The main advantage of the channel-wise approach is that it preserves the spatial structure of degradation. Instead of producing only one average trajectory, the proposed framework produces a set of local degradation trajectories and a profile of degradation acceleration coefficients. Channels with high values of $\alpha_{k}$ can be interpreted as zones of accelerated life consumption. This makes it possible to identify which parts of the belt cross-section enter the refurbishment window first, which parts leave this window, and which zones approach the critical removal condition. As a result, maintenance planning can be based not only on the current amount of damage, but also on the spatial distribution of future life.

The industrial case study illustrates why this distinction matters. The average trend may suggest a moderate and relatively slow degradation process, while a selected channel may already show much faster damage accumulation and earlier threshold exceedance. In such a situation, average-based RUL may overestimate the remaining useful life of the belt section. The proposed minimum local RUL and life-uniformity indicators address this limitation by explicitly comparing the local critical zone with the average cross-sectional trend.

**Figure 10:**
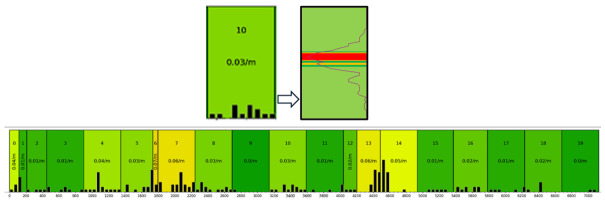
Cross-sectional degradation as a signature of loading asymmetry, shown as a coloured belt segment box with threshold windows and channel profile, in addition to the standard presentation of the belt loop based on average densities and the longitudinal density histogram.

The transverse structure of degradation also has an important diagnostic meaning. A persistent concentration of damage in selected channels may indicate that the belt is not loaded uniformly across its width. This interpretation is supported by studies on transfer chutes and bulk material flow, which show that chute geometry, material trajectory, local impact and shear interaction may strongly affect the distribution of forces acting on the belt [37,38,39]. Therefore, the channel-wise degradation profile can be treated not only as a belt condition indicator, but also as an indirect signature of loading quality. Figure 10 illustrates this concept by linking cross-sectional degradation with loading asymmetry and with the standard longitudinal presentation of belt condition.

This interpretation gives the proposed framework a broader system-level meaning. The belt becomes not only an object being diagnosed, but also a sensor-like recorder of long-term operating conditions in the conveyor system. If a specific transverse zone repeatedly shows accelerated degradation, this may justify corrective actions in the loading or transfer zone. Such actions may include chute adjustment, improvement of material centring, reduction in impact energy or changes in the transfer geometry. The economic justification of these actions should be based on whether the expected increase in belt life exceeds the cost of corrective intervention.

The proposed framework is also consistent with maintenance optimization principles. Decisions concerning refurbishment or removal should not be based only on a final failure threshold. In practice, the user needs to distinguish between normal operation, entry into the refurbishment window, the end of economically justified refurbishment and the critical state. The three-threshold policy proposed in this paper reflects this practical logic and connects channel-wise RUL with specific maintenance actions [17,18].

Several limitations should also be noted. First, the quadratic degradation model is a simplified approximation of damage growth. The limited number of diagnostic scans also restricts robust statistical comparison of alternative degradation models; therefore, future work should validate the proposed framework using longer diagnostic histories and deferred-scan prediction tests. Real degradation trajectories may include quasi-linear phases, sudden damage events, changes in operating conditions or effects of repairs between scans. Second, the threshold values used in the framework require calibration for specific belt types, operating conditions and refurbishment strategies. Third, the economic interpretation proposed in this study is intentionally simplified and does not include downtime costs, risk of unexpected failure, discounting or alternative maintenance schedules. Fourth, the present framework does not yet include direct mechanical validation using DEM or load measurements.

Another limitation of the present model is that degradation parameters are estimated separately for individual channels. This does not mean that neighbouring channels are mechanically independent. On the contrary, transverse zones of the belt may be correlated because damage development is influenced by common operating factors, such as asymmetric loading, material impact, mistracking and transfer point geometry. The proposed compact-zone width indicator partly addresses this issue by distinguishing isolated threshold exceedances from contiguous groups of affected channels. However, a full treatment of spatial correlation would require spatial or spatio-temporal degradation models, which should be considered in future work.

These limitations define natural directions for further research. The model can be extended by introducing uncertainty bounds for $\alpha_{k}$ and RUL values, probabilistic threshold crossing, load-dependent degradation models and combined health indicators based on defect count, defect area and cover wear. Another important direction is the integration of diagnostic data with operating data, such as transported mass, belt speed, loading conditions and transfer point geometry. In the longer term, channel-wise degradation profiles may provide a validation layer for DEM models and digital twins of conveyor loading zones.

Overall, the proposed approach shifts conveyor belt diagnostics from average-based condition assessment towards spatially resolved prognostic decision support. It allows local damage concentration to be quantified, linked with maintenance thresholds and interpreted in terms of life utilization. This makes the method relevant not only for belt replacement and refurbishment planning, but also for diagnosing the quality of conveyor operation as a whole.

## 7. Conclusions

This paper proposed a sensor-based, channel-wise framework for remaining useful life estimation and maintenance decision support for steel-cord conveyor belts. The starting point of the study was the limitation of average damage indicators, which may be insufficient when degradation is spatially non-uniform across the belt width. In such cases, the future usability of a belt section may be governed not by the average cross-sectional condition, but by the most degraded local channel or compact group of channels.

The main contribution of the paper is the formal transition from average belt-section assessment to channel-wise condition and RUL analysis. The belt cross-section was represented as a set of active diagnostic channels, and local damage density was treated as a channel-wise health indicator obtained from consecutive diagnostic scans. This representation preserves the transverse structure of degradation and enables local damage trajectories to be analysed separately.

A simple accelerating degradation model was introduced to estimate local degradation intensity for each channel. The resulting degradation acceleration coefficients provide a transverse profile of life consumption across the belt width. This profile can reveal zones in which damage develops faster than suggested by the average trend. The proposed model is intentionally simple and interpretable, making it suitable for first-level maintenance decision support when only a limited number of diagnostic scans is available.

The framework also introduced a multi-threshold maintenance policy. Instead of defining RUL only as time to a final critical state, three decision thresholds were considered: entry into the refurbishment window, the end of economically justified refurbishment, and the critical removal condition. This makes it possible to distinguish between normal operation, refurbishment planning, controlled operation after the refurbishment window, and removal from service. The proposed indicators, including minimum local RUL, fraction of channels above threshold, compact-zone width and life-uniformity measures, provide a more complete basis for maintenance decisions than a single average RUL value.

The industrial case study demonstrated that a selected channel may degrade much faster than the average belt-section indicator. This confirms that average-based assessment can mask local degradation and may overestimate the remaining useful life of a belt section. The channel-wise approach therefore provides additional diagnostic and prognostic information that is directly relevant to refurbishment and replacement planning.

The study also showed that cross-sectional non-uniformity has an economic interpretation. If one local zone reaches a critical condition early, the entire belt section may be removed before the remaining belt width has fully utilized its potential life. In this sense, local damage concentration reduces life utilization efficiency and may increase the cost per unit operating time. Channel-wise diagnostics can therefore support not only direct belt maintenance decisions, but also corrective actions in loading and transfer zones.

The proposed framework should be treated as a first-level, interpretable model rather than a complete predictive maintenance system. Its main limitations include the simplified quadratic degradation law, the need to calibrate threshold values for specific belt types and operating conditions, and the simplified economic interpretation. Future work should include uncertainty-aware RUL estimation, probabilistic threshold crossing, integration with transported mass and loading-condition data, and validation against mechanical or DEM-based models of transfer zones. Further development may lead to a digital-twin-oriented diagnostic framework in which channel-wise belt degradation profiles are used both for maintenance planning and for assessing the quality of conveyor operation.

Overall, the proposed approach extends conveyor belt diagnostics from average-based condition assessment towards spatially resolved prognostic decision support. By linking sensor-based diagnostic scans, local health indicators, channel-wise RUL, multi-threshold maintenance policy and economic interpretation, it provides a practical foundation for more reliable and cost-aware management of steel-cord conveyor belt life.

## Figures and Tables

**Table 1:** Position of the present study in relation to the most closely related previous works.

Reference	Main Focus of the Previous Work	Limitation Relative to the Present Study	Novelty Introduced in the Present Paper
[2]	Sensor-based diagnostics for conveyor belt condition monitoring and predictive refurbishment.	The study focused on diagnostic support and refurbishment-oriented condition monitoring, but did not formulate channel-wise RUL estimates or a formal multi-threshold policy.	The present paper transforms diagnostic scan data into channel-wise health indicators, RUL estimates and maintenance decision indicators.
[3]	Remaining lifetime forecasting of steel-cord conveyor belts based on regression analysis of damage identified by subsequent DiagBelt scans.	The analysis was performed at a more aggregated belt-section level and did not preserve the transverse channel structure of degradation.	The present paper fits local degradation trajectories separately for diagnostic channels and compares local RUL with average-based RUL.
[12]	Time-dependent growth of damage extents in the steel-cord belt core.	The focus was on damage extent development rather than channel-wise RUL and threshold-based maintenance decisions.	The present paper links local degradation trajectories with multiple maintenance thresholds and decision states.
[16]	Transverse profiles of belt core damage used to interpret loading quality and conveyor operation.	Transverse profiles were used mainly for diagnostic and operational interpretation, not for formal RUL estimation.	The present paper uses transverse channels as local health units for prognostics and decision support.
[40]	Uncertainty analysis in conveyor belt condition assessment using time-based indicators.	The study focused on uncertainty in time-based belt condition indicators and did not formulate channel-wise degradation trajectories, threshold-specific RUL estimates or a multi-threshold maintenance policy.	The present paper extends belt condition assessment towards channel-wise prognostics by transforming transverse diagnostic channels into local health indicators, local RUL estimates, compact-zone measures and maintenance decision states.

**Table 2:** Main variables and channel-wise health indicators used in the proposed framework.

Symbol	Meaning	Interpretation	Unit
L	belt section length	length of the analysed belt section	m
M	number of active channels	number of transverse diagnostic channels	–
$t_{i}$	scan time	time of diagnostic campaign (*i*)	time
$N_{k}(t_{i})$	defect count in channel (*k*)	number of detected defects assigned to channel (*k*)	count
$A_{k}(t_{i})$	damaged area in channel (*k*)	total damaged area assigned to channel (*k*)	area
$d_{k}(t_{i})$	channel damage density	count-based local health indicator	1/m
$a_{k}(t_{i})$	channel area density	area-based local health indicator	area/m
$\bar{d}(t_{i})$	average damage density	average cross-sectional condition	1/m
$d_{max}(t_{i})$	maximum local damage density	most degraded channel	1/m
$C_{d}(t_{i})$	damage concentration coefficient	local peak relative to average condition	–
$\sigma_{d}\left(t_{i}\right)$	standard deviation of channel densities	absolute non-uniformity of transverse profile	1/m
$CV_{d}\left(t_{i}\right)$	coefficient of variation	relative non-uniformity of transverse profile	–
$m_{j}\left(t_{i}\right)$	number of channels above threshold ($T_{j}$)	transverse extent of threshold exceedance	channels
$p_{j}\left(t_{i}\right)$	fraction of channels above threshold ($T_{j}$)	affected fraction of belt width	–
$B_{j}(t_{i})$	compact-zone width above threshold ($T_{j}$)	largest contiguous block of affected channels	channels

**Table 3:** Qualitative sensitivity of maintenance decisions to threshold selection.

Scenario	Threshold Setting	Effect on Estimated RUL	Maintenance Implication
Conservative	Lower T1–T3 values	Shorter RUL to all decision states	Earlier refurbishment or replacement; lower operational risk
Baseline	Nominal calibrated values	Reference RUL values	Standard decision policy
Permissive	Higher T1–T3 values	Longer RUL to all decision states	Later intervention; higher risk of operating close to critical condition

**Table 4:** Operational states and decision meanings in the proposed three-threshold maintenance policy.

State	Operational State	Decision Meaning
A	Normal operation	Continue operation and monitoring
B	Refurbishment window	Plan refurbishment while it is still justified
C	After refurbishment window	Continue only under controlled conditions; plan replacement
D	Critical condition	Remove the belt section from service

**Table 5:** Main characteristics of the industrial diagnostic case study.

Parameter	Value/Description
Conveyor system	Belt conveyor operated in an underground coal mine
Belt type	Steel-cord conveyor belt
Number of analysed belt sections	13
Analysed belt loop length	~5200 m
Belt width	1600 mm
Number of diagnostic scans	4
Total operating time covered by the scans	approximately 150 months
Diagnostic system	DiagBelt+
Transverse channel spacing	25 mm
Number of active diagnostic channels	66–67
Selected channel for local comparison	Channel 26
Primary damage indicator	Number of detected steel-cord core defects
Normalized health indicator	Defect count per metre of analysed belt section
Spatial aggregation used for regression	Defects assigned to transverse diagnostic channels and aggregated over the analysed section length for each scan

## Data Availability

Data is contained within the article.
